# Substance use stigma: A systematic review of measures and their psychometric properties

**DOI:** 10.1016/j.dadr.2024.100237

**Published:** 2024-05-06

**Authors:** Angelica Spata, Ishita Gupta, M. Kati Lear, Karsten Lunze, Jason B. Luoma

**Affiliations:** a Portland Psychotherapy Clinic, Research, and Training Center, Portland, OR, USA; b Dr. Rajendra Prasad Governmental Medical College, Tanda, India; c Boston University Aram V. Chobanian & Edward Avedisian School of Medicine, Boston, MA, USA

**Keywords:** Substance use, Substance use stigma measures, Psychometric, Discrimination, Shame

## Abstract

**Background:**

Instruments to measure substance use stigma are emerging, however little is known regarding their psychometric properties. While research has evolved to view substance use stigma as a context sensitive international phenomenon that is embedded within cultures, validated self-report measures are lacking and comprehensive reviews of the existing measures are extremely limited. In this systematic review of substance use stigma and shame measures, we aim to contextualize results from existing research, lay the groundwork for future measurement development research, and provide a thorough resource for research scientists currently designing studies to measure substance use stigma.

**Methods:**

We searched three databases using Boolean search terms for psychometric evaluations of measures of substance use stigma and shame and evaluated the quality/psychometric properties using an adaptation of the COnsensus-based Standards for the selection of health Measurement Instruments (COSMIN) systematic review guidelines.

**Results:**

We identified 18 measures of substance use stigma. Overall, most measures had minimal psychometric assessments and none of the measures met all domains of the COSMIN measure quality criteria. However, most studies reported satisfactory factor analyses and internal consistency scores.

**Conclusions:**

Most measures of substance use stigma and shame had psychometric assessment across a limited range of criteria and no measures of structural substance use stigma were found. The most reported psychometric properties were structural validity and convergent validity. We suggest future researchers investigate test-retest reliability and cross-cultural validity for existing substance use stigma measures, as well as develop and evaluate novel measures assessing structural stigma of substance use.

## Highlights

• Systematic review identified 18 measures for substance use stigma and shame.
• Most measures lacked evaluation across many psychometric domains.
• Most measures showed satisfactory factor analyses and internal consistency.
• No existing measures for structural substance use stigma were identified.
• Provides guidelines on future measurement development for substance use stigma.

## 1 Introduction

Substance use is widespread, with 50 % of Americans over the age of 12 having used illicit substances in their lifetime (National Center for Drug Abuse Statistics, 2023). Stigma, defined as societal labeling and mistreatment based on perceived differences (Link and Phelan, 2001) leads to a divisive “us” versus “them” dynamic that leads to status loss in a context of power dynamics. Substance use stigma (SUS) involves negative stereotypes and discrimination toward people that use substances, which results in limiting their access to needed resources and impeding wellbeing (Livingston et al., 2012). Stigma is pervasive in society and based out of moral judgments that substance use is bad or wrong (Room, 2005).

SUS significantly hinders treatment and education, adding to the burden carried by people with substance use disorders (Keyes et al., 2010, Kulesza et al., 2013). It limits access to treatment through underfunding of substance use treatment services (Saloner et al., 2014, Zemore et al., 2021, Calabrese et al., 2016, Luoma, 2010) and creates barriers to reintegrating into communities (Neale et al., 2011). Stigma also affects treatment quality, for example leading to denial of effective options like methadone for opiate use disorder (Allen et al., 2019). Stigma promotes social isolation, via family estrangement or avoiding social support due to fear of being stigmatized or discriminated against (Leary et al., 1994, Mateu-Gelabert et al., 2005, Stringer and Baker, 2018). This silence impedes dissemination of accurate information for harm reduction, thereby increasing risk behavior and overdose prevalence. Criminalization of drug use leads to unregulated supplies and barriers to drug testing that could prevent ingestion of unknown or contaminated drugs (Gahlinger, 2004, Sherman et al., 2019).

In addition, people with substance use often have other stigmatized traits and conditions that intersect with SUS, including criminal justice involvement, sex work, or infectious diseases (Stangl et al., 2019), which exacerbates negative outcomes (Vetrova et al., 2021). Families also experience the impacts of secondary or courtesy stigma, specifically blame and shame (Corrigan et al., 2006). Evidence for SUS as a cultural phenomenon can be found in research showing that the forms and intensity of SUS varies across cultures (Simha et al., 2022).

### 1.1 Types of SU stigma

Below we separate SUS into three main types: structural stigma (Knaak et al., 2020, Pachankis et al., 2014, Stangl et al., 2019, Link and Phelan, 2001), social stigma (Palamar et al., 2011), and internalized or self-stigma (Brown-Johnson et al., 2015).

#### 1.1.1 Structural stigma

Structural stigma is defined as policies, institutions, and social structures that isolate and disadvantage stigmatized individuals (Pachankis et al., 2014, Link and Phelan, 2001). Structural SUS is observed in healthcare settings (Knaak et al., 2020, Livingston, 2020), for example in the form of underfunding of substance use treatment relative to other forms of healthcare, resulting in limited access, poor quality of care, or lack of training among health care providers (Stewart et al., 2021). The criminalization of drug use and practices like mandatory minimum sentences results increased incarceration versus treatment of people who use substances and adds additional stigma of top of SUS. Other forms of structural SUS include employment discrimination linked to drug testing, housing discrimination, exclusionary health insurance policies, and social service exclusions. Structural stigma also impedes effective harm reduction policies such as safe consumption sites (Hawk et al., 2015, Marlatt and Witkiewitz, 2010, Ahrens, 2020). Structural stigma disproportionately affects individuals from intersectionally oppressed groups; for example, a person of color with a substance use disorder is likely to experience more structural stigma than a white person with a substance use disorder (Farahmand et al., 2020).

#### 1.1.2 Social stigma

Social or public stigma is defined as the public’s opinion and behavior towards people that use substances and includes stereotyping, emotional reactions, and overt discrimination (Yang et al., 2017). This can occur both covertly and overtly. For example, the public is often punitive toward people with SUDs, thinking they are blameworthy (Johnson-Kwochka et al., 2021). Stigma also manifests in social distance, defined as the willingness or reluctance of stigmatizing groups to be in proximity to, interact, or communicate with individuals that use substances, perhaps due to perceived dangerousness (Brown, 2011). Additionally, social stigma may involve stigma-related affect, which is the emotional response to individuals using substances (Brown, 2011). Social stigma also includes enacted stigma, which refers to discriminatory behavior that serves to dehumanize, distance, or devalue, such as through covert micro aggressions (Grigsby et al., 2018) or through more overt distancing, disadvantaging, or even physical violence (Pawlukewicz, 2004, Smith et al., 2020, Smith et al., 2016).

#### 1.1.3 Internalized stigma or self-stigma

Internalized or self-stigma refers to an individual’s stigmatizing attitudes towards themselves for their substance use and the resulting behavioral consequences (Matthews et al., 2017, Chen et al., 2020). It involves awareness of stereotypes (Chang et al., 2020) that when applied to the oneself can result in self-esteem decrement, self-devaluation, or shame (Smith et al., 2020, Yang et al., 2019). Substance-use related shame, in particular, is considered to be a core emotion involved in internalized stigma (Luoma et al., 2012). Internalized or self-stigma also involves reactions to anticipated stigma, which refers to an expectation of being stigmatized, often at the hands of family members, employers, or healthcare providers (Smith et al., 2020, Smith et al., 2016). The effects of these internalized beliefs and resulting emotions and perceptions may be further exacerbated by the person’s coping attempts, such as avoidance of social situations or internal avoidance behaviors (Luoma et al., 2013).

In contrast to rich literature on SUS and importance of this topic, existing measures of SUS are lacking and have little psychometric support (Kwakep epse Semegni et al., 2021). In order to advance the study of SUS stigma, this systematic review aimed to identify all existing measures of SUS as well as all existing measures of substance-use related shame, since this construct is so closely linked to SUS. We sought to evaluate each measure’s psychometric properties, provide suggestions on how to improve future measurement, and inspire researchers to create new measures for SUS that are more culturally appropriate.

## 2 Method

### 2.1 Search strategy

Our comprehensive search strategy was based on adapted recommendations from the COnsensus-based Standards for the selection of health Measurement INstruments (COSMIN; Prinsen et al., 2018). The search began with a search of PsychInfo, PubMed, and WebofScience databases for psychometric evaluations of SUS measures using the following Boolean search terms (“stigma” or “shame”) AND (“substance use” or “addiction” or “drug use” or “alcohol use” or “drug abuse” or “substance abuse” or “alcohol abuse”) AND (“psychometrics” or “measure development”).

Records were evaluated against the following inclusion criteria. We included any peer-reviewed, published paper that reported a psychometric evaluation of a measure of SUS or substance use-related shame, as that is an aspect of internalized stigma. We included measures of SUS across any type of substance. Studies were excluded if they focused on more general measures of shame and stigma that did not specifically relate to substance use. We did not include studies that did not include a psychometric evaluation. Articles that fit the scope were required to be full-text empirical articles (not case reports, commentaries, or dissertations) written in English and with adult study samples.

### 2.2 Methodological quality assessment of identified measures

We evaluated measures across the 9 COSMIN domains: development validity, content validity, structural validity, internal consistency, cross-cultural validity/measurement invariance, test-retest reliability, construct validity, and responsiveness. Findings for each domain were summarized descriptively rather than using the full COSMIN rating process as it was determined that the nascent level of research in this area would result in nearly uniform poor ratings and little variability in coded validity and reliability of the scales. We report Cronbach’s alphas of .70 as ‘adequate’, .80 were reported as ‘good’, and alphas .90 and above as ‘high.’ Two authors were involved in the screening and quality assessment processes and any disagreements were resolved via discussion with a senior author. No ethics committee approval was required for this study.

## 3 Results

### 3.1 Overview of measures and studies

**Fig. 1:**
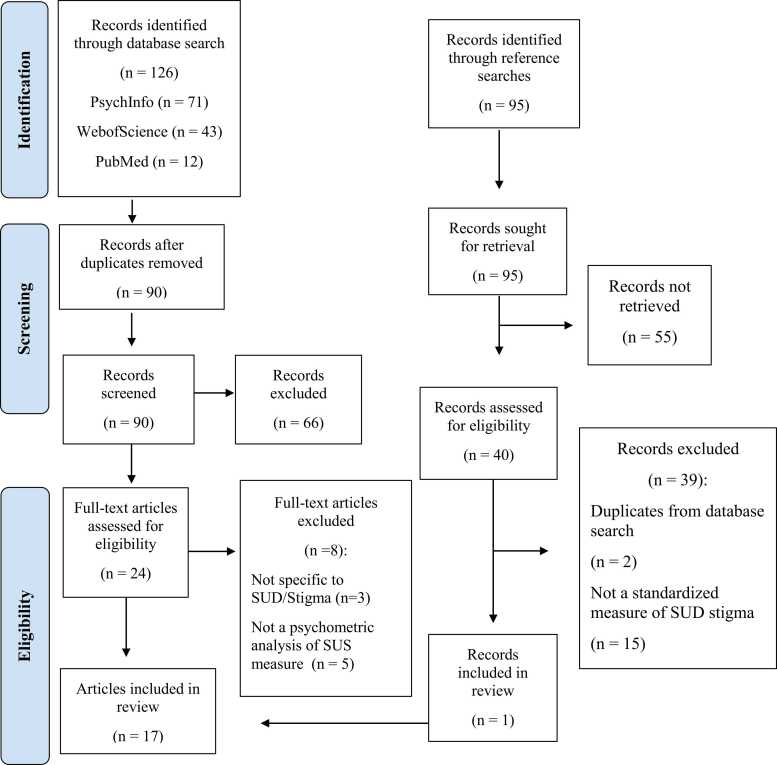
Prisma flow diagram.

Records identified through the search process are detailed in the PRISMA flow diagram (Moher et al., 2009) in Fig. 1. Initial database searches generated a total of 126 papers. Screening of titles and abstract yielded a total of 25 potential papers for which full text copies were obtained and reviewed by AS and IG. We consulted a third rater (JBL) when there was ambiguity or disagreement on whether an article met inclusion/exclusion criteria. We identified an additional study through hand-searching reference lists of selected papers. In total, 17 articles with 20 unique samples were included. Of those studies that reported gender, 51 percent were women and 49 % men. Samples were drawn from people in substance use treatment (55 % of samples), students (18 % of samples), community or a convenience samples (18 % of samples), and other settings (9 %). Detailed sample characteristics are outlined in Table 1. We evaluated 2 measures of substance use related shame, 7 of internalized stigma, and 12 of social stigma, with 2 measures accounting for both internalized stigma and social stigma. No measures of structural stigma were located for evaluation. Basic information about the included measures can be found in Table 2.

**Table 1:** Summary of study characteristics.

Authors	Population Characteristics					Country	Measure of Substance Use Stigma	Measurement Properties Assessed
	*N*	Study Population	Age M(*SD)*	Gender Makeup	Race/ Ethnicity			
Brown (2011)								
Sample	565	University students (UG)	18.6 (1.0)	69 % w; 31 % m	Caucasian 96 %	USA	SDS-SU, DS-SU, AS-SU	IC, CVV, DV
Brown-Johnson et al. (2015)								
Sample	956	People who smoke from a tobacco treatment clinical trial in the Bay Area	ND	466 w; 490 m	African American 218 (23 %); Asian/Pacific Islander 44 (5 %); White 454 (48 %); Hispanic 95 (10 %); Other 145 (15 %)	USA	ISSI	SV, IC, CVV, DV
Chang et al. (2020)								
Sample	300	Patients with SUD in Tainan, Taiwan.	45.22 (9.99)	45 w; 255 m	ND	Taiwan	PSAS	SV, MI, IC
Subsample 1	100	Patients with primary heroin use disorder	49.16 (7.09)	14 % w; 86 % m	ND	Taiwan	PSAS	MI
Subsample 2	100	Patients with primary amphetamine use disorder	42.64 (9.31)	17 % w; 83 % m	ND	Taiwan	PSAS	MI
Subsample 3	100	Patients with primary alcohol use disorder	45.97 (10.70)	13 % w; 87 % m	ND	Taiwan	PSAS	MI
Chen et al. (2020)								
Sample	784	Patients in drug rehabilitation for methamphetamine use	31.59 (7.16)	108 w; 676 m	ND	China	CSDS, CSS	SV, IC, MI
Subsample 1	603	Voluntary patients in drug rehabilitation for methamphetamine use	31.47 (6.88)	29 w; 574 m	ND	China	CSDS, CSS	SV, MI
Subsample 2	181	Compulsory patients in drug rehabilitation for methamphetamine use	31.98 (8.03)	79 w; 102 m	ND	China	CSDS, CSS	SV, MI
Glass et al. (2013)								
Sample	34,373	Combined subsamples 1–5. U.S. Adults from Wave 2 of the NESARC	ND	52 % w; 48 % m	White 71 %; Black 11 %; Native American 2 %; Asian 4 %; Hispanic 12 %	USA	PAS	SV, CVV, DV
Subsample 1	4634	Lifetime alcohol abstainers	ND	ND	White 55 %; Black 16 %, Native American 3 %, Asian 10 %, Hispanic 17 %	USA	PAS	DV
Subsample 2	18,459	Individuals who drink alcohol, but have never met AUD criteria	ND	ND	White 70 %; Black 12 %; Native American 2 %, Asian 5 %, Hispanic 12 %	USA	PAS	DV
Subsample 3	6640	Individuals in AUD recovery	ND	ND	White 80 %, Black 7 %, Native American 3 %, Asian 2 %, Hispanic 8 %	USA	PAS	DV
Subsample 4	2365	Individuals who currently have AUD	ND	ND	White 75 %, Black 10 %, Native American 2 %, Asian 2 %, Hispanic 10 %	USA	PAS	DV
Subsample 5	2288	Individuals who have received treatment for AUD	ND	ND	White 75 %, Black 10 %, Native American 4 %, Asian 1 %, Hispanic 10 %	USA	PAS	DV
Grigsby et al. (2018)								
Sample	90	Patients in residential treatment for substance use	32.92 (10.82)	54 % w; 46 % m	Non-Hispanic White 56 %, Hispanic 21 %, African American 16 %, Asian/Pacific Islander 7 %	USA	MCS	RL, SV, CVV, IC
Johnson-Kwochka et al. (2021)								
Sample	304	MTurk participants	37.2 (10.97)	117 w; 187 m	White or Caucasian 252 (82.9 %), Hispanic 39 (12.8 %)	USA	AQ-SUD	CV, SV, IC, CVV, DV
Luoma et al. (2010)								
Sample	252	Participants in SUD treatment	30.5 (9.95)	106 w; 145 m	Native American 4 %, Asian/Pacific Islander 1 %, African American 4 %, Caucasian 79 %, Other 7 %, NR 6 %, Latino 12 %	USA	PSAS	CV, IC, SV, CVV, DV
Luoma et al. (2013)								
Sample 1	17	Residential Treatment program for SUD	ND	ND	ND	USA	SASSS	CV
Sample 2	352	Residential and Outpatients for SUD	31.1 (10.2)	141 w; 210 m	American Indian/Alaskan Native 14 (4 %), Asian/Pacific Islander 3 (9 %), Black 15 (4 %), White 283 (80 %), Other 23 (7 %), NR 14 (4 %); Mexican 26 (7 %), Other Hispanic 13 (7 %), Not of Hispanic Origin 147 (41 %), NR 163 (46 %)	USA	SASSS	CV, SV, CVV, IC, CRV
Palamar et al. (2011)								
Sample 1	700	Convenience internet sample	29.32 (9.2)	69 % w; 31 % m	White 67 %, Black 7 %, Hispanic/Latino 12 %, Asian/Pacific Islander 9 %, Other 4 %	USA	SDUS, PPS	CV
Sample 2	1048	People recruited on the street in the Manhattan area of New York	20.31 (1.9)	54 % w; 47 % m	White 44 %, Black 14 %, Hispanic/Latino 17 %, Asian/Pacific Islander 18 %, Other 8 %	USA	SDUS, PPS	SV, CVV, IC
Pawlukewicz (2004)								
Sample	75	Outpatients for SUD	ND	50 % w, 50 % m	ND	USA	AAII	RL, CVV, CRV
Subsample 1	15	Outpatients for SUD	ND	ND	ND	USA	AAII	IC
Subsample 2	7	Outpatients for SUD	ND	ND	ND	USA	AAII	CV
Smith et al. (2016)								
Sample	178	Combined subsample 1 & subsample 2	43.51 (11.33)	84 w; 94 m	Hispanic 60 (34 %), non-Hispanic Black 46 (26 %), non-Hispanic White 64 (36 %), non-Hispanic Other 8 (5 %)	USA	SU-SMS	CV, SV, IC, DV, CVV
Subsample 1	93	Patients receiving methadone maintenance therapy	38.10 (10.21)	46 w; 47 m	Hispanic 13 (14 %), non-Hispanic Black 13 (14 %), non-Hispanic White 63 (68 %), non-Hispanic Other 4 (4 %)	USA	SU-SMS	
Subsample 2	85	HIV positive patients receiving HIV and/or buprenorphine therapy	49.43 (9.34)	38 w; 47 m	Hispanic 47 (55 %), non-Hispanic Black 33 (39 %), non-Hispanic White 1 (1 %), non-Hispanic Other 4 (5 %)	USA	SU-SMS	
Smith et al. (2020)								
Sample	93	Patients in methadone maintenance in the NE United States	38.10 (10.21)	46 w; 47 m	Latino 13 (14 %), Non-Hispanic Black 13 (14 %), Non-Hispanic White 63 (68 %), Non-Hispanic Other 4 (4 %)	USA	MMT-SMS	CV, SV, CVV, DV, IC
Stockton et al. (2021)								
Sample	400	Methadone maintenance patients in Vietnam	41.3 (7.2)	3 w; 397 m	ND	Vietnam	PSAS	SV, DV, IC
Treeby et al. (2020)								
Sample 1	293	Community members and university students	22.25 (7.87)	213 w, 80 m	White 92 %, Asian 4 %, Other or Mixed ethnicity 4 %	Australia	PODS-S	CV, IC
Sample 2	429	Community members and university students	23.87 (8.97)	311 w, 109 m	White 92 %, Asian 4 %, Black 1 %, Other or mixed ethnicity 3 %	Australia	PODS-S	SV, IC, RL, CVV, DV
Tuliao and Holyoak (2019)								
Sample	791	Undergraduate students (UG)	20.80 (2.47)	590 w, 201 m	White 557 (70 %), Latinx 129 (16 %), African American 39 (5 %), Biracial 40 (4 %), Asian American 20 (3 %), Native American 4 (1 %)	USA	PSAS	SV, IC, CVV
Yang et al. (2019)								
Sample	387	Patients in inpatient opioid withdrawal program	33.1 (8.40)	26 % w; 74 % m	Hispanic 39 (10 %) White 320 (82 %), Black 12 (3 %), Other 55 (14 %)	USA	BOSS	CV, SV, IC, DV, CVV

*Note.* ND = Not Described; NR = No Response; UG = Undergraduate students specified; G = graduate students specified; SUD = Substance Use Disorder; AUD = Alcohol Use Disorder; NESARC: National Epidemiologic Survey on Alcohol and Related Conditions; CV: content validity; CVV: convergent validity; CRV: criterion validity; IC: internal consistency; MI: measurement invariance; RL: reliability; RS: responsiveness; SV: structural validity; DV: Discriminant Validity; SDS-SU: Social Distance Scale for Substance Users; DS-SU: Dangerousness Scale for Substance Users; AS-SU: Affect Scale for Substance Users; ISSI: Internalized Stigma of Smoking Inventory; PSPS-TV: Taiwan Version of the Perceived Stigma toward People who use Substances; CSDS: Chen et al. Social Distance Scale; CSS: Chen et al. Stigma Scale; PAS: Perceived Alcohol Stigma Scale; MCS: Micro-Condescension Scale; AQ-SUD; Attribution Questionnaire-Substance Use Disorders; PSAS: Perceived Stigma of Addiction Scale; SASSS: Substance Abuse Self-Stigma Scale; AAII: Acceptance of an Alcoholic Identity Instrument; SDUS: Stigma of Drug Users Scale; SU-SMS: Substance Use Stigma Mechanisms Scale; MMT-SMS: Methadone Maintenance Treatment Stigma Mechanisms Scale; PODS-S: Perceptions of Drinking Scale – Shame Subscale; BOSS: Brief Opioid Stigma Scale.

**Table 2:** Description of questionnaires and subscales.

Measure	Intended Population	Construct Measured	Scale Structure	Number of Items	Response Options	Scoring Method
AAII-Shame Subscale	Individuals with AUD	Shame	Total Score; unidimensional	11	4-point scale (1=Never – 4=Always)	Mean of all items
AQ-SUD	General Public	Attitudes Toward SUD	Total score with 4 subscales: Negative emotions, assessment of responsibility, lack of empathy, and social disengagement	18	9-point scale (1=Not at all – 9=Very likely)	Items summed
AS-SU	General Public	Stigma Related Affect	Total score	10	7-point scale	Mean of all items
BOSS	Populations that currently or previously used opioids	Self-Stigma (Stereotype Awareness, Stereotype Agreement, Self-esteem decrement)	3 subscales: Aware, Agree, Harm	12	5-point scale (1=Strongly Disagree – 5=Strongly Agree)	Mean of all items
CSDS	Individuals with SUD	Social Distance	Total score	5	4-point scale (1=Definitely Willing To – 5=Definitely Unwilling To)	Mean of all items
CSS	Individuals with SUD	Personal & perceived stigma	2 subscales: personal stigma, perceived stigma	18	5-point scale (1=Strongly Agree – 5=Strongly Disagree)	Mean of all items
DS-SU	General Public	Perceived Dangerousness	Total score	7	7-point scale (Strongly Agree – Strongly Disagree)	Mean of all items
ISSI	Individuals that smoke	Internalized Stigma	Total score with 3 subscales: self-stigma, felt stigma, and discrimination	8	4-point scale (1=Strongly Disagree – 4=Strongly Agree)	Mean of all items
MCS	Individuals in SUD treatment	Micro Stigmas	Total score with 1 factor	12	5-point scale (1=Not at all true – 5=Extremely true)	Items summed
MMT-SMS	Individuals receiving methadone maintenance treatment	Enacted, Anticipated, and Internalized Stigma	Total Score with 3 subscales: Anticipated Stigma, Enacted Stigma, Internalized Stigma	25	5-point scale (1=Never – 5=Very Often)	Mean of all items
PAS	Individuals that drink alcohol	Perceived Stigma	Total score with 1 factor	12	6-point scale (1=Strongly Agree – 6=Strongly Disagree)	Items summed
PODS-S	Individuals that drink alcohol	Alcohol use-related shame	Shame Subscale from larger 4 subscale measure	5	5-point scale (1=Strongly Disagree – 5 Strongly Agree)	Items summed
PPS	General Public	Perceived Stigma	Total score; unidimensional	10	5-point scale (1=Strongly Disagree – 5 (Strongly Agree)	
PSAS	Individuals with SUD	Perceived Stigma	Total factor with 1 factor	6–9	4-point scale (1=Strongly Disagree – 4=Strongly Agree)	Items summed
SASSS	General Public	Self-stigma	Total score with 3 subscales: self-devaluation, fear of enacted stigma, stigma avoidance and values disengagement	8	5-point scale (1=Never or almost never – 5=Very often)	NR
SDUS	General Public	Stigmatization of people who use substances	Total score; unidimensional	7	5-point scale (1=Strongly Disagree – 5=Strongly Agree)	NR
SDS-SU	General Public	Social Distance	Total score	7	4-point scale (Definitely Willing – Definitely Unwilling)	Mean of all items
SU-SMS	Individuals that use substances	Enacted, Anticipated, and Internalized Stigma	3 subscales:	18	5-point scale (Anchors NR)	Mean of all items

*Note.* Designation as scale indicates the measure is a standalone assessment of substance use stigma, while designation of subscale indicates that the substance use stigma scale reviewed is a subscale in a broader measure; AAII = Acceptance of an Alcoholic Identity Instrument; AQ-SUD = Attribution Questionnaire - Substance Use Disorders; AS-SU = Affect Scale for Substance Users; AUD = Alcohol Use Disorder; BOSS = Brief Opioid Stigma Scale; CSDS = Chen et al. Social Distance Scale; CSS = Chen et al. Stigma Scale; DS-SU = Dangerousness Scale for Substance Users; ISSI = Internalized Stigma of Smoking Inventory; MCS = Micro-condescension Scale; MMT-SMS = Methadone Maintenance Treatment Stigma Mechanisms Scale; NR = Not reported; PAS = Perceived Alcohol Stigma Scale; PODS-S = Perceptions of Drinking Scale, Shame Subscale; PPS = Perceived Public Stigma; PSAS = Perceived Stigma of Addiction Scale; SASSS = Substance Abuse Self Stigma Scale; SDUS = Stigma of Drug Users Scale; SDS-SU = Social Distance Scale for Substance Users; SU-SMS = Substance Use Stigma Mechanisms Scale; SUD = Substance Use Disorders.

### 3.2 Characteristics and performance of individual measures

#### 3.2.1 Substance-use related shame

##### 3.2.1.1 Acceptance of an Alcoholic Identity Instrument (AAII) – shame subscale

The AAII was assessed in one sample and is purported to measure “acceptance of an alcoholic identity among ‘abstinent alcoholics’” (Pawlukewicz, 2004, p. 1) and is intended to be used with people in recovery from alcohol use disorder. It has 70 items with 6 subscales, including an 11-item subscale designed to assess shame related to alcoholism. The scale was developed based on clinical experience and reports of common experiences during recovery. Items were reviewed by clinicians experienced in chemical dependency, professionals employed in SUD treatment, and people in recovery from alcohol use disorder. Participants were further encouraged to provide comments during assessments. No factor analysis was performed to assess the structural validity of the scale. Alpha for the shame subscale was good in a sample of 75 people in outpatient SUD treatment. Thirty-day test-retest correlation was .85. Limited convergent validity data was provided, with the shame subscale correlating with measures of emotional responding and reactions to various substance use related situations. It was not correlated with a denial subscale. No information was reported on structural validity, cross-cultural validity/measurement invariance, or responsiveness.

##### 3.2.1.2 Perceptions of Drinking Scale – Shame Subscale (PODS-S)

The PODS was assessed in one sample (Treeby et al., 2020) and has a 5-item shame subscale that is part of a larger 17 item scale assessing alcohol use-related shame and guilt. It is intended to be used with populations that use alcohol. A large item pool was screened by trainee psychologists and people with problematic alcohol use and then reduced to the final set via an exploratory factor analysis (EFA) which resulted in four factors, one of which is the shame subscale. The measure was then tested in a sample of community members and university students where a confirmatory factor analysis (CFA) model confirmed the four-factor structure. The shame subscale had a high internal consistency and a test-retest reliability of.90. Evidence for convergent validity came from small-to-moderate correlations with risky alcohol behavior and alcohol related consequences and small correlations with a range of other measures. No information was available on cross-cultural validity/measurement invariance or responsiveness.

#### 3.2.2 Internalized Stigma

##### 3.2.2.1 Brief Opioid Stigma Scale (BOSS)

The BOSS was assessed in one sample (Yang et al., 2019) and measures self-stigma as comprised of stereotype awareness, stereotype agreement, and self-esteem decrement surrounding opioid dependence as perceived by people who use opioids and is intended to be used by people who have used opioids. It was based on the Self-Stigma of Mental Illness Scale (Corrigan et al., 2006) and includes 12 items assessing 4 opioid related stereotypes across 3 subscales. The measure was evaluated in patients from an inpatient opioid withdrawal program. An EFA supported the hypothesized three factor solution. Internal consistency for the three subscales were low to inadequate. Although it is not stated directly, it appears the subscales are meant to be used independently and not as a unitary scale as an overall alpha was not reported. Convergent validity analyses came from associations with measures of self-esteem, mental health, and depression. No information was provided on content validity, cross-cultural validity/measurement invariance, test-retest reliability, and responsiveness.

##### 3.2.2.2 Internalized Stigma of Smoking Inventory (ISSI)

The ISSI was assessed in one sample (Brown-Johnson et al., 2015), measures stigma among pepole who smoke, and has 8 items with 3 subscales, labeled discrimination experiences (2 items), self-stigma (3 items), and felt-stigma (3 items) and is intended to be used with people who smoke. The scale was adapted from the Internalized Stigma of Mental Illness (ISMI) measure (Boyd Ritsher et al., 2003) by changing item content, excluding items that were assessed as nonrelevant, and by obtaining input from experts. The scale was evaluated in a sample of people in a smoking cessation trial. An EFA showed three factors that were confirmed via CFA. Subscales had adequate to good levels of internal consistency and the overall scale had good internal consistency. Construct validity was assessed using regression and showed that variance in all subscales was associated with other experiences of stigma and readiness to quit. No information was provided on cross-cultural validity/measurement invariance, test-retest reliability, and responsiveness.

##### 3.2.2.3 Micro-condescension Scale (MCS)

The MCS was assessed in one sample (Grigsby et al., 2018) and assesses perceptions of micro level stigmatizing events classified as condescension—an attitude of pity or of being viewed as having lower social status. The 12-item scale is intended to be used among people in substance use treatment. Items were developed in meetings with people in recovery and items were piloted for readability and comprehensibility. An EFA showed a one factor solution that had a high internal consistency. Test-retest reliability was calculated through Intraclass Correlation Coefficients, ranging from .37 to .74. Convergent validity analyses showed strong correlations with a perceived devaluation scale and moderate correlations with self-awareness and impulsivity. No information was provided on cross-cultural validity/measurement invariance or responsiveness.

##### 3.2.2.4 Methadone Maintenance Treatment Stigma Mechanisms Scale (MMT-SMS)

The MMT-SMS was assessed in one sample (Smith et al., 2020) and has 25 items with 3 higher order subscales measuring anticipated stigma (9 items), enacted stigma (9 items), and internalized stigma (7 items) with three stigma sources (family, health care workers, and employers) nested within these subscales as lower order scales. Items are rated on a 5-point Likert scale. The measure is intended to be used with populations receiving methadone treatment. Items were developed with input from people who use substances and literature review and then piloted with a small group of people with substance use histories via cognitive interview. The measure was then tested in a sample of patients in methadone maintenance. A hierarchical CFA confirmed seven factors, some of which were nested inside the 3 higher order factors. Internal consistency was high to moderate across all three higher order factors. MMT-SMS scale scores were associated with SUS scale scores. MMT-SMS subscale scores were also associated with readiness to reduce drug use or engage in safer injecting behavior, greater heroin use and withdrawal and urine toxicology results. No information was provided on cross-cultural validity/measurement invariance, test-retest reliability, or responsiveness.

##### 3.2.2.5 Substance Abuse Self Stigma Scale (SASSS)

The SASSS was assessed in two samples (Luoma et al., 2013) and is a 40 item scale with four subscales measuring self-devaluation (8 items), fear of enacted stigma (9 items), stigma avoidance (13 items), and values disengagement (10 items). This measure was based on a theory about the behavioral effects of stigma (Luoma et al., 2012) that resulted in the latter two subscales assessing the effects of stigma-related emotions and cognitions on behavior. It is intended to be used with people who use substances. Measure items were informed by literature review that identified common stereotypes around people who use substances, an item generation and refinement phase, and review by people in SUD treatment and SUD treatment experts. In a sample of people in SUD treatment, an EFA found the four factors described above. All four subscales, as well as the full scale, had good internal consistency. As evidence of criterion validity, those who had no substance use in the last 30 days had lower scores on several of the subscales. Evidence for convergent validity came from small-to-moderate correlations with a variety of other measures including perceived stigma, psychological flexibility, shame, and self-concealment. No information was provided on cross-cultural validity/measurement invariance, or responsiveness.

##### 3.2.2.6 Substance Use Stigma Mechanisms Scale (SU-SMS)

The SU-SMS was assessed in one sample (Smith et al., 2016) and is an 18-item scale that measures experiences of enacted stigma (6 items), anticipated stigma (6 items), and internalized stigma (6 items) with two stigma sources (family, health care workers). The measure is intended to be used with populations that use substances. Items were developed with input from researchers who study SUD and literature review and then piloted with a small group of people with substance use histories and HIV with a cognitive interview. The measure was then tested in a sample of patients in methadone maintenance and an HIV+ sample. A hierarchical CFA confirmed five factors, with the source factors nesting inside the content factors. Internal consistency was high across all three higher order factors. SU-SMS scale scores showed small to moderate correlations across a variety of other variables such as depression, other forms of stigma, and problematic substance use. No information was provided on cross-cultural validity/measurement invariance, test-retest reliability, or responsiveness.

#### 3.2.3 Social stigma

##### 3.2.3.1 Attribution Questionnaire - Substance Use Disorders (AQ-SUD)

The AQ-SUD was assessed in one sample (Johnson-Kwochka et al., 2021) and measures attitudes toward a character in drug-specific vignettes (i.e., an adolescent with opioid, marijuana, stimulant, and alcohol use) and is based on the attribution model of mental illness stigma (Corrigan et al., 2003). The AQ-SUD was adapted from the Attribution Questionnaire-27 (Corrigan et al., 2003) and has 18 items. The measure is intended to be used with the general public to assess stigma-related attitudes toward adolescents who use substances. An original 27-item version was revised to an 18-item version via a combination of a CFA, EFA, removing some items due to irrelevance, and writing new items. An EFA conducted in an adult MTurk sample resulted in a four-factor structure labeled negative emotions, assessment of responsibility, lack of empathy, and social disengagement. All four subscales had good or high alphas. Some of the subscales demonstrated convergent validity with measures of social disengagement, familiarity with SUDs, current stress, and belief in biogenetic causes of SUD. Stigma scores also varied across different forms of substance use. No information was reported on content validity, cross-cultural validity/measurement invariance, test-retest reliability, or responsiveness.

##### 3.2.3.2 Affect Scale for Substance Users (AS-SU)

The AS-SU was assessed in one sample (Brown, 2011) and assesses emotions related to interacting with a person “with a substance use problem” by responding to 10 pairs of bipolar dimensions (e.g., “relaxed-tense”). The measure is intended to be used by general public and was adapted from the Affect Scale (Penn et al., 1994), which originally focused on mental illness. The measure showed high internal consistency in a university student sample. Convergent validity analyses showed a moderate correlation with a measure of affect toward mental illness. No information was available on content validity, structural validity, cross-cultural validity/measurement invariance, test-retest reliability, responsiveness.

##### 3.2.3.3 Chen et al., Social Distance Scale (CSDS)

The CSDS was assessed in one sample (Chen et al., 2020) and is made up of five items and measures willingness to have contact with people that use substances. The measure was adapted from the Social Distance Scale (Link et al., 1999) for mental illness to reflect attitudes toward people who use substances. The measure was tested in a Chinese sample in methamphetamine treatment centers and is intended to be completed by people who use substances but appears appropriate for use with other populations. The scale was translated to Chinese from English and back-translated. A CFA confirmed a unidimensional scale and they found it had good internal consistency based on Cronbach’s alpha. They also demonstrated measurement invariance between their samples of voluntary and compulsory patients at the methamphetamine treatment centers. The authors did not report analyses for content validity, test-retest reliability, convergent validity, or responsiveness.

##### 3.2.3.4 Chen et al. Stigma Scale (CSS)

The CSS was assessed in one sample (Chen et al., 2020) and measures personal stigma defined as an individual’s own stigmatizing attitudes toward people who use substances and perceived stigma defined as the perception of the prevalence of stigmatizing attitudes and behaviors in society as a whole. The measure has 18 items across two 9-item subscales. The measure was tested in a Chinese sample in methamphetamine treatment centers and is intended to be completed by people who use substances but appears appropriate for use with other populations. The measure was adapted from the Depression Stigma Scale (Griffiths et al., 2004) and translated to Chinese from English and back-translated. A CFA confirmed a two-factor structure and Cronbach’s alphas were adequate to moderate across the two subscales. The scale was shown to be invariant across multiple treatment centers. No information was provided on content validity, test-retest reliability, convergent validity, or responsiveness.

##### 3.2.3.5 Dangerousness Scale for Substance Users (DS-SU)

The DS-SU was assessed in one sample (Brown, 2011) and assesses the perceived dangerousness of “individuals with a previous substance use problem” using 7 items. The measure is intended to be used with the general public and was adapted from the Social Distance Scale (Link et al., 1987), which originally focused on mental illness. The measure showed acceptable internal consistency adequate in a university student sample. Convergent validity analyses showed a moderate correlation with a measure of perceived dangerousness toward people with mental illness. No information was available on content validity, structural validity, cross-cultural validity/measurement invariance, test-retest reliability, or responsiveness.

##### 3.2.3.6 Perceived Alcohol Stigma Scale (PAS)

The PAS was assessed in one sample (Glass et al., 2013) and assesses perceived stigma, defined as the awareness of public stigma, and has 12 items. The PAS was adapted from the Perceived Devaluation-Discrimination Scale (Link et al., 1987) and is intended to be used with populations that drink alcohol. The measure was assessed using a general population sample. CFA models resulted in a one factor solution. Convergent validity found that higher perceived stigma was associated with lower social support and lower social network involvement. Invariance analyses suggested that the measure did not function as well with lifetime abstainers. No information was available on content validity, internal consistency, cross-cultural validity/measurement invariance, or responsiveness.

##### 3.2.3.7 Perceived Public Stigma (PPS)

The PPS was assessed in two samples (Palamar et al., 2011) and is a 10-item measure of perceived public stigma that allows researchers to assess public stigma toward specific drugs based on inserting the name of the drug into the appropriate place in the scale. This measure is intended to be used by the general public. Items were developed through a literature search and from previous scales, reviewed by two experts, tested on a pilot sample, and further revised and reviewed by a panel of experts. The pilot sample was an online community sample and the second was a sample of emerging adults. A CFA and EFA confirmed that the items were distinct from the Stigma of Drug Users Scale (see below; Palamar et al., 2011), which was also developed in the same study. Reliability of the scale across 5 types of drugs was in the acceptable to good range. The scale demonstrated limited evidence of construct validity through correlations with some forms of recent drug use but not others and with some demographic variables. No information was available on content validity, cross-cultural validity/measurement invariance, test-retest reliability, or responsiveness.

##### 3.2.3.8 Perceived Stigma of Addiction Scale (PSAS)

The PSAS was assessed in four samples (Stockton et al., 2021, Tuliao and Holyoak, 2019, Chang et al., 2020, Luoma et al., 2010) and was originally developed by Luoma et al. (2010) as an 8-item measure of perceived stigma intended to be used with the general public. The measure was adapted from the Link et al. (1997) discrimination-devaluation measure to refer to “someone who has been treated for substance use” instead of mental illness. A group of experts in addictions rated the original items for quality and face validity and the measure was tested in a sample of outpatient SUD treatment. An EFA resulted in a one-factor scale. This version of the scale had an acceptable internal consistency and demonstrated convergent validity through moderate correlations with other stigma-related measures. A second study of the PSAS (Tuliao et al., 2019) used an undergraduate US population and removed one item based on a CFA, resulting in a 7-item version of the scale that had a good internal consistency. This 7-item version also demonstrated some convergent validity through small correlations with a variety of variables such as help seeking intentions and self-concealment. A third study (Stockton et al., 2021) tested the 8-item version in a sample of Vietnamese methadone patients, presumably after translating it to Vietnamese, though this is not clear in the publication. The original 8-item version showed acceptable internal consistency as did a reduced 6-item version that they created as a result of a CFA model. Another version of this scale was created in Taiwanese (Chang et al., 2020) and added an additional item to create a 9-item version. This version was tested in multiple samples of people in SUD treatment. A CFA confirmed a one-factor structure. Other analyses showed the measure was invariant across different groups. Convergent validity was demonstrated by correlations with measures of internalized stigma, self-esteem, and depression. No information was available on test-retest reliability or responsiveness.

##### 3.2.3.9 Social Distance Scale for Substance Users (SDS-SU)

The SDS-SU was assessed in one sample (Brown, 2011) and assesses a person’s willingness to interact with a person “with a substance use problem” across seven situations resulting in seven items. The measure is intended to be used by general public and was adapted from the Dangerousness Scale (Link et al., 1987), which originally focused on mental illness. The measure showed good internal consistency in a university student sample. Convergent validity analyses showed a small-to-moderate correlation with a measure of social distance from people with mental illness. No information was available on content validity, structural validity, cross-cultural validity/measurement invariance, test-retest reliability, or responsiveness.

##### 3.2.3.10 Stigma of Drug Users Scale (SDUS)

The SDUS was assessed in two samples (Palamar et al., 2011) and is a 10-item measure of stigmatization of people who use substances, defined as the negative social response to those who use illicit drugs and uses a 5-point Likert scale. This scale it intended to be used with the public and allows researchers to assess attitudes toward specific drugs by inserting the name of the drug into the appropriate place in the scale. Items were co-developed with the PSS and tested in the same samples. A CFA and EFA confirmed that the items were distinct from the PSS which was also developed in the same study. Internal reliability of the scale across 5 types of drugs was in the good range. The scale demonstrated some weak construct validity through correlations with some forms of recent drug use and with demographic variables. No information was available on content validity, cross-cultural validity/measurement invariance, test-retest reliability, or responsiveness.

## 4 Discussion

This systematic review summarized the measurement properties of published measures of substance use-related stigma and shame. Although recent papers have demonstrated the adverse effects stigma has on stigmatized communities, the measurement of SUS has lagged. A previous published review of SUS measures included six measures (Kwakep epse Semegni et al., 2021) while we were able to uncover sixteen SUS measures and two substance use-related shame measures, resulting in a more comprehensive review. No measures of structural stigma were found.

Most measures were assessed on only a limited number of measurement properties. In particular, most measures lacked an evaluation of cross-cultural validity/measurement invariance, responsiveness, and test-retest reliability. Thus, relatively little is known about the reliability and validity of these measures across varying samples, how responsive these measures are to intervention, or how stable these constructs are across time. Most studies assessed structural validity through factor analyses and reported on internal consistency, but few used confirmatory approaches (e.g., CFA or IRT analyses) to provide high quality evidence for their structural validity. Also, most studies reported adequate alpha levels. Many studies included some measures of convergent validity, but often these measures selected seemed of limited relevance to stigma and therefore the evidence for convergent validity across many of these measures is weak. The majority of measures (i.e., the BOSS, ISSI, AQ-SUD, AS-SU, CSS, DS-SU, PAS, PSAS, and SDS-SU) utilized a notably weak development process wherein item content was altered from existing mental illness stigma measures with sometimes new items being added without assessing their quality. This process is notably flawed in terms of content validity as the content of stigma is known to vary between substance use and mental illness (Corrigan et al., 2009, Luoma et al., 2013), which will presumably result in content validity problems when important content domains or constructs are not assessed.

Only one measure was psychometrically studied in more than one study, the PSAS (Luoma et al., 2010). This measure was assessed across three additional samples beyond the original and generally confirmed its factor structure. Unfortunately, each study added or removed items, so it is difficult to compare results across the different studies. In general, although we realize the need to keep assessments short that are administered to participants, it would be helpful if researchers reported results related to already existing measures before adding or removing items so that comparability across studies can be assessed.

In terms of future research, more studies are needed to examine whether existing psychometric findings replicate in new samples. In particular, evidence for convergent validity is sorely needed as concurrent measures were often not relevant to the construct of stigma. Studies that incorporate multiple stigma measures would be useful to examine how different measurement approaches result in distinguishable empirical findings. Much more research is needed to see if these measures are responsive to intervention. Further, more SUS measures need to be assessed for cross-cultural validity with only 3 of the 18 current SUS instruments having any evidence (PSAS, CSDS, CSS; Chang et al., 2020; Chen et al., 2020).

Those wishing to study individual level interventions to reduce substance use-related shame or stigma face the choice of appropriate outcome measures (Luoma et al., 2023). For example, one recent review of interventions to reduce SUS among health care providers found studies using a wide range of measures, some of which had limited psychometric evaluation (Magnan et al., 2024). Certain stigma manifestations are unlikely to be reduced by individual-level interventions. For example, perceived stigma could increase as a stigma intervention might prompt higher awareness of stigma in society. Similarly, measures of experiences with enacted stigma, such as in the SU-SMS (Smith et al., 2016) or MMT-SMS (Smith et al., 2020) are not likely to be reduced due to intervention as these are reports of past events. Instead, more proximal measures of personal stigmatizing attitudes, stigma-related coping, or personal experiences with stigmatizing behavior are most likely to be useful as outcome measurements. For example, interventions for public stigma might benefit from assessing desire for social distance, as measured in the SDS-SU (Brown, 2011), or negative social responses toward people who use drugs, as measured by the SDUS (Palamar et al., 2011), rather than perceived stigma. Similarly, for measures targeting internalized stigma or substance use related shame, measures such as the shame subscale from the PODS (Treeby et al., 2020) or the self-devaluation or stigma coping scales from the SASSS (Luoma et al., 2013) are more likely to be responsive to intervention. Regardless, response to intervention has yet to be assessed in any psychometric study we found.

While using COSMIN domains was a strength of this review, we did not engage in the full COSMIN rating process, as the consistently low measurement quality assessment in most studies would have resulted in little variability in ratings across measures and nearly uniform ratings of poor performance. The lack of a standardized rating process may have introduced bias into the review. It’s also possible that our review strategy or search terms may have missed articles that should have been included.

Our literature review also yielded several stigma measures that have been created but not yet evaluated psychometrically. We included these in an online supplementary document (https://osf.io/56vpq) so that researchers can consider them for further psychometric development. This included measures that have been utilized across multiple studies despite lack of psychometric evaluation (e.g., the Internalized Stigma of Substance Abuse; Luoma et al., 2011) and measures of novel stigma-related constructs via an experimental task (Ashford et al., 2019). Measures were also adapted to various cultural and language contexts such as Brazil (da Silveira et al., 2018) or Egypt (Mohammed Ali, 2019) or sub populations such as incarcerated women (Moore et al., 2020), adolescents (Adlaf et al., 2009) but not psychometrically studied.

This systematic review considerably expands the understanding of the current state of measurement of substance use-related stigma and shame. Despite recent growth in the number of measures, the findings highlight serious shortcomings in psychometric evaluation. The various dimensions of stigma were unevenly represented and some, like structural stigma, were not assessed by any measure we could find. Looking forward, it is critical for future researchers to more rigorously evaluate existing measures and to use more valid approaches when evaluating new measures. In addition, since stigma is a multifaceted construct, researchers need to carefully select the right measure of stigma for their study context. Furthermore, investigations into how these measures respond to interventions is vital to validate their responsiveness to change and their use in informing stigma reduction strategies. In sum, this review outlines the need for further rigorous measure development work and psychometric evaluation to better assess the multifaceted construct of substance use stigma.

## Funding

This work was funded by internal funding through Portland Psychotherapy’s social-research business model.

## CRediT authorship contribution statement

**Ishita Gupta:** Writing – original draft, Data curation, Conceptualization. **Angelica Spata:** Writing – review & editing, Writing – original draft, Methodology, Formal analysis, Data curation, Conceptualization. **Jason B. Luoma:** Writing – review & editing, Writing – original draft, Supervision, Methodology, Formal analysis, Data curation, Conceptualization. **Karsten Lunze:** Writing – review & editing, Conceptualization. **M. Kati Lear:** Writing – review & editing, Writing – original draft, Methodology, Conceptualization.

## Declaration of Competing Interest

The authors have no conflicts of interest to declare.
